# Supplementary material for: Effect of Wild Strawberry Tree and Hawthorn Extracts Fortification on Functional, Physicochemical, Microbiological, and Sensory Properties of Yogurt
Source: Foods. 2023 Sep 5;12(18):3332. doi: 10.3390/foods12183332 (PMC10528895; doi:10.3390/foods12183332)
Supplement: Supplementary file 1 [file foods-12-03332-s001.zip › foods-2574340-supplementary.pdf]

Effect of Wild Strawberry Tree and Hawthorn Extracts Fortification on Functional, Physicochemical, Microbiological and Sensory Properties of Yogurt

Teresa Herrera, Maite Iriondo-DeHond, Ana Ramos Sanz, Ana Isabel Bautista, Eugenio Miguel *

Área de Investigación Agroalimentaria. Instituto Madrileño de Investigación y Desarrollo Rural, Agrario y Alimentario (IMIDRA), 28805 Alcalá de Henares, Spain

* Correspondence: eugenio.miguel@madrid.org; Tel.: 0034 91 887 94 10

Note: The data presented in this study are available on request from the corresponding author. The data are not publicly available due to the authors are "in preparation" for another manuscript.

Table of contents

Table S1. Total phenolic content (TPC) (mg GAE/g extract) antioxidant capacity (µmol TE/g extract) and inhibitory of digestive enzymes of Strawberry tree extracts (STE) and Hawthorn extracts (HE).

Table S2. Results of total counts of mesophilic aerobic bacteria (PCA media), enterococci (VRBG media), coliforms (VRBL media), fecal enterococci (KF media) and mold and yeast (PDA media) of Strawberry-tree extracts (STE) and Hawthorn extracts (HE) (log ufc/g of extract).

Table S1.

	Strawberry tree				*Hawthorn*			
	STE	*STE 1h RT*	*STE 24 h RT*	*STE 1h 60 ºC*	*HE*	*HE 1h RT*	*HE 24 h RT*	*HE 1h 60 ºC*
TPC (mg GAE/g extract)	9.27 ± 0.47[a]	12.73 ± 0.62[ab]	12.23 ± 0.34[ab]	17.93 ± 4.17[cd]	19.67 ± 0.38[cd]	17.99 ± 1.50[cd]	15.55 ± 1.08[bc]	22.01 ± 1.16[d]
Antioxidant capacity								
ABTS (µmol TE/g extract)	227.40 ± 22.49[a]	596.39 ± 83.30[bc]	572.45 ± 26.95[ab]	573.24 ± 10.31[cd]	443.92 ± 12.75[bc]	615.94 ± 72.34[cd]	556.52 ± 102.95[bcd]	731.00 ± 32.29[d]
ORAC (µmol TE/g extract)	158.72 ± 14.40[a]	172.14 ± 14.68[a]	214.00 ± 11.37[a]	190.39 ± 7.35[a]	528.35 ± 20.50[c]	396.95 ± 66.45[b]	353.29 ± 34.75[b]	625.94 ± 27.60[d]
Antidiabetic properties								
α-Amylase inhibition (%)	4.21 ± 0.42[a]	5.39 ± 0.96[a]	5.46 ± 0.65[a]	7.47 ± 1.06[b]	4.88 ± 0.68[a]	5.47 ± 0.18[a]	6.02 ± 0.45[ab]	4.66 ± 0.58[a]
α-Glucosidase inhibition (%)	80.60 ± 1.67[b]	55.28 ± 5.62[a]	51.65 ± 2.52[a]	54.07 ± 9.90[a]	77.83 ± 2.84[b]	56.97± 7.59[a]	53.31 ± 2.86[a]	57.92 ± 0.92[a]
α-Glucosidase IC_{50} (mg/mL)	4.40 ± 0.26	9.00 ± 1.38	9.13 ± 0.48	7.38 ± 0.36	5.13 ± 1.26	8.20 ± 1.27	9.24 ± 0.66	8.01 ± 0.27
Lipase inhibition (%)	59.32 ± 3.51[a]	84.77 ± 9.84[b]	64.08 ± 0.28[a]	97.79 ± 15.74[b]	91.74 ± 2.36[b]	93.75± 4.47[b]	102.57 ± 1.14[b]	91.01 ± 2.88[b]
Lipase IC_{50} (mg/mL)	13.71 ± 4.87[c]	12.46 ± 1.09[abc]	11.85 ± 0.34[bc]	8.22 ± 0.68[abc]	3.40 ± 0.60[a]	3.47 ± 0.43[ab]	4.32 ± 0.14[abc]	3.75 ± 0.44[a]
Antihypertensive properties								
ECA inhibition (%)	14.21 ± 3.24[a]	14.64 ± 4.31[a]	15.71 ± 5.72[a]	18.43 ± 4.88[a]	18.94 ± 3.16[a]	22.72 ± 3.76[a]	21.20 ± 4.23[a]	17.09 ± 4.89[a]

Results are reported as mean ± SD (*n*=3). Different letters within the same column denote statistically significant differences ($p \leq 0.05$).
Inhibition of digestive enzymes were analysed at 10 mg/mL of extracts.

Table S2.

Extracts	Mesophilic aerobic bacteria (log ufc/g of extract)	Enterococci (log ufc/g of extract)	Coliforms (log ufc/g of extract)	Fecal enterococci (log ufc/g of extract)	Mold and yeast (log ufc/g of extract)
STE	2.33 ± 1.94[ab]	n.d.	n.d.	n.d.	2.00 ± 1.79[abc]
STE 1h RT	3.00 ± 0.00[ab]	1.00 ± 1.79[a]	n.d.	n.d.	n.d.
STE 1h 60 ºc	2,00 ± 1.79[a]	n.d.	n.d.	n.d.	n.d.
STE 24h RT	4.30 ± 1.22[ab]	4.33 ± 0.20[bc]	2.67 ± 2.42[ab]	n.d.	4.67 ± 0.78[cd]
HE	4.00 ± 0.12[ab]	3.67 ± 0.39[b]	3.67 ± 0.24[bc]	n.d.	4.33 ± 0.24[bcd]
HE 1h RT	1.30 ± 2.54[a]	n.d.	1.33 ± 2.41[ab]	n.d.	2.67 ± 2.35[abcd]
HE 1h 60 ºc	4.00 ± 0.56[ab]	n.d.	n.d.	n.d.	n.d.
HE 24h RT	6.00 ± 0.00[b]	6.00 ± 0.00[c]	6.00 ± 0.70[c]	n.d.	6.00 ± 0.04[d]

Results are reported as mean ± SD (n=3). Different letters within the same column denote statistically significant differences ($p \leq 0.05$).
n.d.: no detected

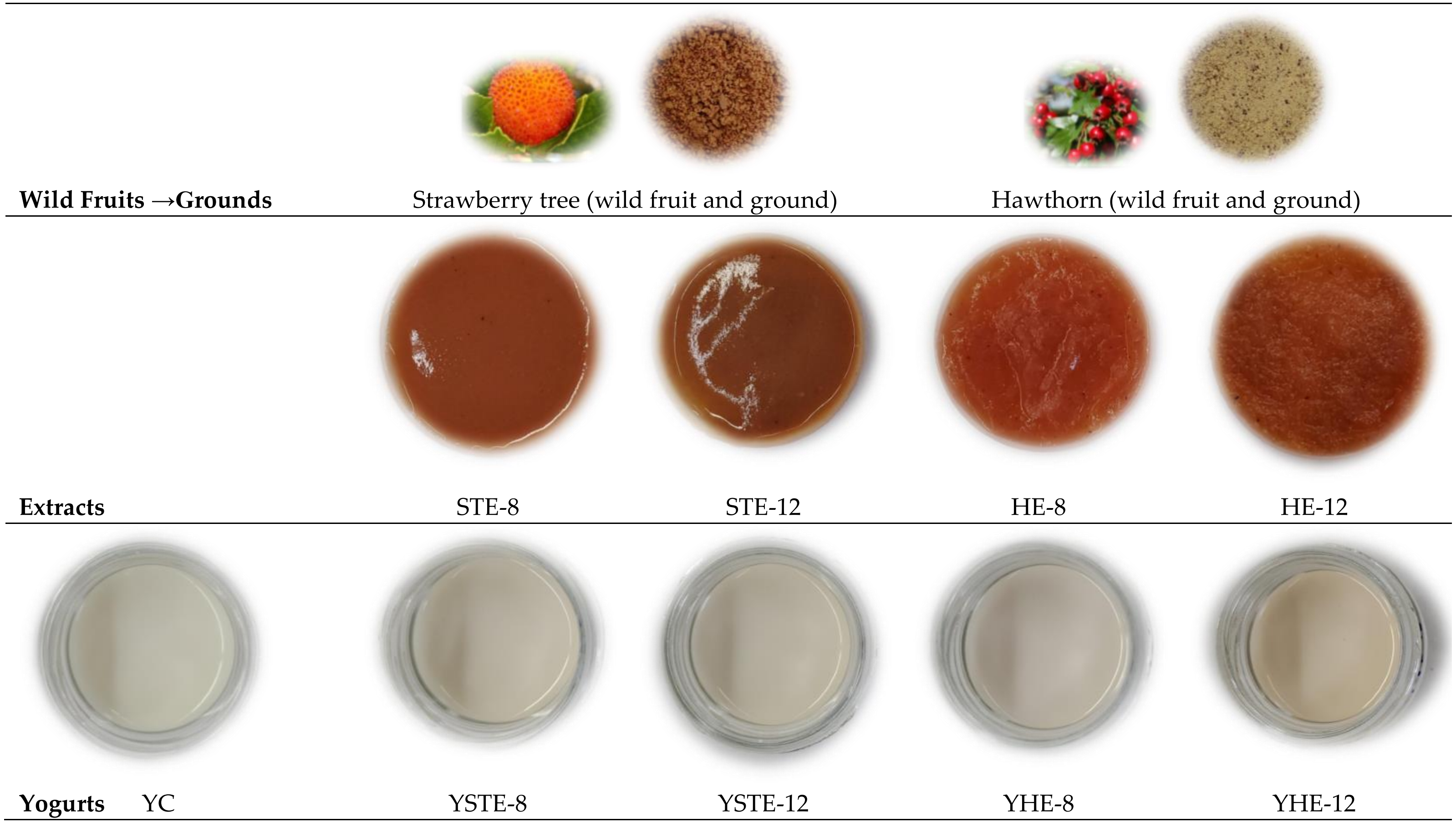

Figure S1. Images of wild fruits, grounds, extracts and yogurts from strawberry tree and hawthorn that were employed in this study.
